# Which Task Characteristics Do Students Rely on When They Evaluate Their Abilities to Solve Linear Function Tasks? – A Task-Specific Assessment of Self-Efficacy

**DOI:** 10.3389/fpsyg.2021.596901

**Published:** 2021-03-12

**Authors:** Katharina Siefer, Timo Leuders, Andreas Obersteiner

**Affiliations:** ^1^University of Education Freiburg, Freiburg im Breisgau, Germany; ^2^University of Education Ludwigsburg, Ludwigsburg, Germany

**Keywords:** self-efficacy, linear functions, confirmatory factor analysis, assessment, self-report

## Abstract

Self-efficacy is an important predictor of learning and achievement. By definition, self-efficacy requires a task-specific assessment, in which students are asked to evaluate whether they can solve concrete tasks. An underlying assumption in previous research into such assessments was that self-efficacy is a one-dimensional construct. However, empirical evidence for this assumption is lacking, and research on students’ *performance* suggests that it depends on various task characteristics (e.g., the representational format). The present study explores the potential multi-dimensionality of self-efficacy in the topic of linear functions. More specifically, we investigate how three task characteristics – (1) the *representational* format, (2) embedding in a *real-life context*, or (3) the required *operation* – are related to students’ self-efficacy. We asked 8th and 9th graders (*N* = 376) to evaluate their self-efficacy on specific linear function tasks which systematically varied along the three dimensions of task characteristics. Using confirmatory factor analysis, we found that a two-dimensional model which includes the task characteristic of real-life context (i.e., with vs. without a real-life context) fitted the data better than other two-dimensional models or a one-dimensional model. These results suggest that self-efficacy with linear functions is empirically separable with respect to tasks with vs. without a real-life context. This means that in their self-evaluation of linear function tasks students particularly rely on whether or not the linear function task is embedded in a real-life context. This study highlights the fact that even within a specific content domain students’ self-efficacy can be considered a multi-dimensional construct.

## Introduction

Self-efficacy is an important predictor of school learning and it is closely linked to performance (Bandura, 1977; Valentine et al., 2004; Zarch and Kadivar, 2006; Klassen and Usher, 2010; Honicke and Broadbent, 2016; Talsma et al., 2018). Self-efficacy can be understood as “a situational or problem-specific assessment of an individual’s confidence in her or his ability to successfully perform or accomplish a particular task or problem” (Hackett and Betz, 1989, p. 262). In line with this definition, Bandura (2006, 1977) recommended assessing self-efficacy in a task-specific way. One way of conducting task-specific assessments is to confront individuals with concrete mathematical tasks and ask them how well they think they are able to solve them. Another way is to provide an individual with a (more abstract) description of a type of mathematical task (instead of presenting the tasks themselves) and ask them to evaluate their abilities. The former approach seems preferable because it requires less abstraction. However, the caveat to this approach is that it is unclear which characteristic of the tasks presented students will actually rely on when evaluating their own abilities. Previous studies that used task-specific assessments of self-efficacy in mathematics often do so without considering the potential impact of a student’s interpretation of different task characteristics (Kranzler and Pajares, 1997; Krawitz and Schukajlow, 2018). An implicit assumption of such a task-specific definition and assessment is that self-efficacy is a one-dimensional construct. However, it is largely unclear whether and in which cases this is a valid assumption. There are few studies (Street et al., 2017) which have addressed the empirical separability of self-efficacy dimensions in mathematics, and there is no study in the domain of linear functions. The present study investigates the way in which students’ self-efficacy regarding linear functions depends on task characteristics. We chose the mathematical topic of linear functions because in this domain research has identified task characteristics that actually affect performance (Leinhardt et al., 1990; Bayrhuber et al., 2010; Schukajlow et al., 2012; Bock et al., 2015). It is also a key topic in the mathematics curriculum in all grades. As a general goal, this study aims to combine a domain-specific, mathematics-educational perspective with a more psychological perspective on self-efficacy.

### Self-Efficacy

Bandura defined self-efficacy as “people’s beliefs about their capabilities to produce designated levels of performance” (Bandura, 1994, p. 2). In comparison to other related constructs, such as the academic self-concept, self-efficacy is related to a specific activity for solving a problem rather than a general evaluation of one’s own competence (Marsh et al., 2018). Self-concept is often conceptualized in a broader way than self-efficacy and it encompasses the entire system of beliefs about oneself and one’s self-evaluation (Shavelson et al., 1976), which includes knowledge about oneself, personal qualities, competences, interests, feelings, and behavior (Rosenberg, 1979). Marsh et al. (2018) distinguished between both constructs on a theoretical and empirical basis using a sample of *N* = 3350 students. These authors suggest three main distinctions between self-efficacy and self-concept: first, the relation to which the assessment of self-concept or self-efficacy takes place (self-efficacy stands in relation to one’s individual self, self-concept in relation to a social group); second, the temporal orientation of the prediction (self-efficacy is related to the future, self-concept is related to the past); and third, the evaluation or description of the constructs (self-efficacy seems more a description of one’s own abilities whereas self-concept has a higher abstraction). In our study we focus on the construct of self-efficacy.

**FIGURE 1:**
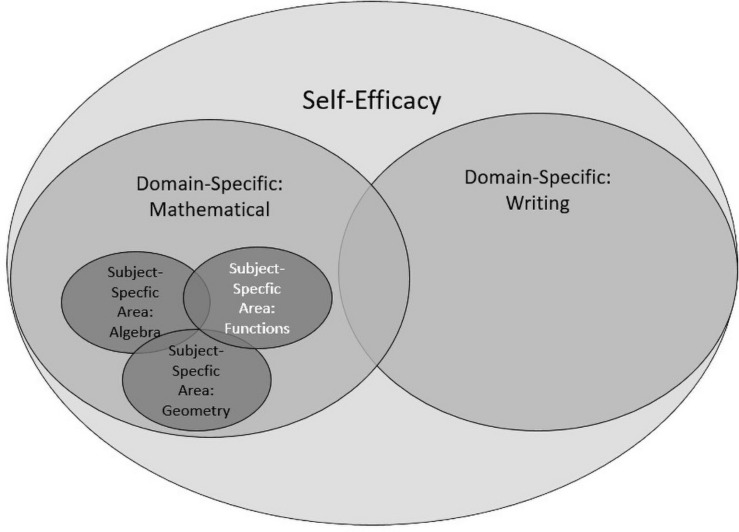
Levels of self-efficacy with varying subject specificity.

The concept of self-efficacy is not uniformly used in the literature, which can make interpretations of empirical findings difficult (for an overview see Bong and Skaalvik, 2003; Ferla et al., 2009; Marsh et al., 2018). Roughly, the literature on self-efficacy has differentiated between varying levels of self-efficacy in respect to specificity (Bandura, 2006; Honicke and Broadbent, 2016; Marsh et al., 2018) (see Figure 1 for an overview). At the first and most general level, self-efficacy (largest circle in Figure 1) represents one’s confidence in one’s ability to successfully perform at school, such as in classroom discourse, seatwork, homework or in tests (Mittag et al., 2002). An example of an instrument that assesses self-efficacy at this general level is the frequently used survey by Jerusalem and Satow (1999). An example of a question in their instrument is: “I can solve difficult tasks if I pay attention in class.”

At the second, domain-specific, level, self-efficacy depends on certain domains such as school mathematics or writing (two medium-sized circles) (Lewis et al., 2012; Marsh et al., 2018). One example is the frequently used Mathematics Self-Efficacy Scale (MSES) scale by Betz and Hackett (1983) as well as Kranzler and Pajares (1997) with items like: “I feel confident enough to ask questions in my mathematics class.” Other than in the scales of Jerusalem and Satow (1999) described above, items in this instrument explicitly refer to the domain of mathematics.

The MSES scales of Betz and Hackett (1983) also include a sub-scale with concrete tasks, which is a characteristic of the third level of self-efficacy. At this most specific level, self-efficacy is considered in a specific subject area (the smallest circles) such as geometry, algebra (Hackett and Betz, 1989) or functions (Siefer et al., 2020), which are all areas of mathematics. Students have to evaluate their abilities for solving specific tasks. Typically, students are presented with specific tasks and have to indicate for each whether they think they have the ability to solve it successfully. The major difference to the domain-specific level is the use of concrete tasks.

These diverse conceptualizations demonstrate that a theoretical clarification and empirical investigation on the operationalization of self-efficacy seems worthwhile (Pajares and Kranzler, 1995; Bandura, 2006). Bandura (1977) highlighted that there is not only one correct way to measure self-efficacy, but the assessment of self-efficacy should depend on the context. He emphasizes the necessity to be attentive to a variety of demands within a given domain or task. In fact, Bandura (1997) clearly states that, “in developing efficacy scales, researchers must draw on conceptual analysis and expert knowledge of what it takes to succeed in a given pursuit” (p. 43). Therefore, it seems important that instruments take into account the context of what (content) the students are taught in school.

Empirical studies find that the correlations between self-efficacy, when assessed at different levels (task-specific and domain-specific assessment), and self-concept are far from perfect, suggesting that different kinds of self-efficacy assessments may actually tap into different underlying constructs. For example, Marsh et al. (2018) found that the correlation between domain-specific self-efficacy (or “generalized self-efficacy,” p. 21) in mathematics and task-specific self-efficacy (or “test-related self-efficacy,” p. 22) was moderate to high (*r* = 0.58). Moreover, domain-specific self-efficacy in mathematics correlated more strongly with mathematical self-concept than task-specific self-efficacy. Accordingly, domain-specific self-efficacy seems to be related more closely to a student’s self-concept than task-specific self-efficacy. In conclusion, to assess self-efficacy one should use an operationalization that is linked tightly to the theoretical conceptualization of self-efficacy as a task-specific construct. In the following, we briefly describe different ways to assess self-efficacy in a task-specific way.

#### Task-Specific Assessment of Self-Efficacy

Some studies used verbal descriptions of tasks to assess self-efficacy, which may be considered an “indirect assessment” (Bofah and Hannula, 2011; Dreher et al., 2020). For example, Dreher et al. (2020) used statements like “I’m sure I can solve tasks with graphs” to assess self-efficacy for graphs.

Another way of using a task-specific assessment of self-efficacy is to present students a concrete mathematical task and to ask them how confident they feel about being able to solve this task (Siefer et al., 2020). Such an assessment may be considered as a more “direct assessment”; indeed, there are some studies which have used such a form of assessment. The frequently used Mathematics Self-Efficacy Scale (MSES) by Hackett and Betz (1989) includes 18 concrete mathematical problems from the fields of arithmetic, algebra and geometry based on Dowling (1978). The reliability of the whole scale of mathematical self-efficacy was high (*Cronbach’s alpha* = 0.92). Yet the authors did not analyze the dimensionality of self-efficacy further with respect to the different content areas of arithmetic, algebra and geometry. Moreover, the rationale for choosing tasks from these content areas remains unclear and we do not know to what extent specific task characteristics may have affected students’ self-evaluation. Another example of a task-specific assessment is the study by Bonne and Johnston (2016), who used 10 arithmetic problems (reliability not reported).

The studies described above utilized specific tasks and showed good reliabilities. The studies all relied on the assumption that self-efficacy is a one-dimensional construct, and they did not investigate its potential multi-dimensionality. There are few studies focusing on the potential multi-dimensionality of self-efficacy. One example is the study by Bruning et al. (2013), which focused on general self-efficacy in writing with middle school students (*N* = 696). The authors used confirmatory factor analysis to show that in their sample self-efficacy was a three-dimensional construct. The three dimensions in writing could be classified as idea generation, observing conventions and self-regulation. However, in their study, Bruning et al. (2013) did not use concrete tasks to represent the three dimensions. In contrast, Street et al. (2017) used concrete tasks of a mathematics performance test with 756 Norwegian 5th, 8th, and 9th graders. They also used confirmatory factor analysis to show that a multi-dimensional model fitted the data best. Of course, their results depended on the tasks used and the models tested. The dimensions of confirmatory factor analysis were structured according to the level of difficulty (easy, medium, difficult) in the performance test. However, other task characteristics were not considered. A requirement for the validity of a direct assessment is that tasks are selected carefully in order to cover all relevant parts of the target domain or topic. Students are then supposed to be able to rely on all important task characteristics which may actually affect their performance. It is possible that the same task characteristics which had been shown to affect performance are also relevant to students’ evaluation of their self-efficacy. However, other task characteristics which have not been considered yet may systematically play a role too. In order to explore systematically such influences in the present study, we address the question of dimensionality of self-efficacy within a particular mathematical context: linear functions. Linear functions are a central topic in school mathematics.

### Characteristics of Linear Functions

Students develop self-efficacy in mathematics through solving specific mathematical tasks. Students may associate their success or failure in working with these tasks with specific task characteristics (e.g., a specific representation) or, more generally, with the complete content area of the tasks (e.g., functions). The role of task characteristics is well studied in the domain of linear functions. Linear functions are also an interesting topic to study because they are a key concept within the domain of mathematics and within school curricula at all ages (Vollrath, 1989; Elia et al., 2008). Understanding functions is relevant in real-world contexts (Van Dooren and Greer, 2010), and it is correlated with abstract thinking as well as with performance in other mathematical topics like problem solving (Leuders et al., 2017; Krawitz and Schukajlow, 2018). Most importantly, research on linear functions has identified the challenges that students have with respect to specific task characteristics. In the following we describe three task characteristics which research has identified as being challenging for many students. These task characteristics are also typically addressed in the mathematics classroom in line with curricula and standards for school mathematics [e.g., National Governors Association Center for Best Practices & Council of Chief State School Officers, 2010; Ministerium für Kultus, Jugend und Sport in Baden-Württemberg., 2016].

A first task characteristic when working with functions is the representational format. Representation in the field of linear functions includes graphs, tables and algebraic terms as well as situational-verbal representations. Solving function tasks often requires working with these representations. Therefore, this characteristic includes the ability to use different forms of representation (Leinhardt et al., 1990; Ainsworth, 1999; Duval, 2006; Elia et al., 2008). There is broad empirical evidence that the type of representation is relevant for students’ competencies related to functions (including their knowledge, their abilities, their preferences, etc.). Elia et al. (2008) argue that the ability to deal with representations is indispensable for a deep understanding of the concept of functions. Bayrhuber et al. (2010) assessed problem-solving abilities of 872 13–14 year-olds when working with different representations of linear functions. The authors showed with latent class analysis that students have different profiles with respect to graphical, numerical and situational-verbal representations. Studies which investigated students’ preferences (Keller and Hirsch, 1998) found that students tend to prefer certain representations depending on the context of the task. Furthermore, Acevedo Nistal et al. (2013) showed in their think-aloud study that a student’s (age 14–16) justification of his/her choice for using a specific representation (graph, table, term) for solving function tasks could be classified by several dimensions, namely: task-related, subject-related, context-related and representation-related justifications. The result of the study documented a large number of subject-related justifications (operationalized as justifications where students’ subject characteristics influenced the choice itself), but participants hardly ever gave reasons for these subject-related justifications. Students often voiced personal preferences, yet what these preferences were based on remained unclear. It seems possible that students have a particular confidence in their abilities when dealing with, for example, the representational format of the graph. In summary, all these studies show that the representational aspect of task characteristics is very important and influences performance.

A second task characteristic when working with functions relates to the context of the task. Students have to understand the specific context for linear function tasks because these tasks are often embedded in a real-life context (Schukajlow et al., 2012; Van Dooren et al., 2018). For example, determining the slope of a function in an intra-mathematical task may be easier than interpreting the meaning of the slope in the context of a mountain hike (Bell and Janvier, 1981). Bock et al. (2015) showed that students had far fewer problems using a negative slope in an intra-mathematical context than in an extra-mathematical context. In contrast, Schukajlow et al. (2012) used self-reports and different tasks in the context of linear functions as well as Pythagoras’s theorem. The tasks were classified as intra-mathematical tasks, word problems and modeling problems. The authors found no significant difference in self-efficacy between intra-mathematical tasks, word problems and modeling problems. The results are not in line with other research findings by Van Dooren et al. (2018), for example, who found that the context of a task played an important role. A possible reason relates to the method of assessment via self-report or the mix of the two topics of linear functions and Pythagoras’s theorem. The mix of these two topics does not offer the chance to have different self-reports for multiple topics.

A third important task characteristic when solving function tasks is the specific operation that needs to be carried out. For example, tasks may ask students to *describe* the type of a graph or table, *draw* a graph from a given equation, *interpret* a table or *complete* a table with given information (Nitsch et al., 2015; Rolfes et al., 2018). These types of operations may also affect how difficult a problem is. For example, tasks which require creating a graph may be perceived as more difficult than tasks which require reading off a point on a graph. There are only few empirical studies which focus on the task characteristic of “operations” with regard to linear functions. One rare example is the study by Rolfes et al. (2018), which focused on the interaction between different kinds of operation and different representations (graph, table, bar charts) when solving function tasks. The study found that retrieving information is easier with a table than with a graph, and that interpreting growth is easier with a graph than with a table.

In summary, theoretical considerations and empirical evidence suggest that the three task characteristics of representation, context and operation may affect student performance on linear function tasks. We therefore expect that students rely on different task characteristics when they evaluate their own abilities. Consequently, a student’s self-efficacy may be affected by some task characteristics, but not necessarily by all three task characteristics to the same extent.

### The Present Study

In the literature, self-efficacy is assumed to be domain- and task-specific. Accordingly, when students are asked about their self-efficacy in a certain area, they should be presented with concrete tasks. However, in such an assessment using concrete tasks, it is not clear which task characteristics students actually consider in their evaluation. We therefore explore the relevance of different task characteristics in linear function tasks: their representation, the context and the required operation. This selection of task characteristics resulted from previous studies on performance in linear functions. With respect to the representation, we distinguish between graphs and tables. Regarding the context, we consider intra-mathematical and extra-mathematical tasks. With respect to the operation, we distinguish between creating (a graph or a table) and reading off information (from a graph or a table).

Using a task-specific assessment of self-efficacy, we were interested in whether in our data self-efficacy is a one-dimensional construct or whether it is a multi-dimensional one along the dimensions of the task characteristics of representation (graph/table), context (intra-mathematical/extra-mathematical) and/or operation (create/read). We assume that students rely on one or more of these task characteristics to evaluate their abilities. However, the current state of research on self-efficacy does not allow us to make predictions about which task characteristics may play a more or less prominent role for students. Therefore with respect to multi-dimensionality, we were interested in the question of which characteristics (representation, context, operation) best represented the data derived from students’ task-specific self-evaluation.

The specific research questions were:

(1) Is self-efficacy (assessed via task-specific self-evaluation) a one-dimensional construct or a multi-dimensional construct along the three selected dimensions of task characteristics?
(2) Which task characteristics do students rely on most in their evaluation?

## Materials and Methods

### Participants

The Ministry of Education in Germany responsible approved the study. Invitations were sent to medium-track secondary schools (German “Realschule”) in southern Germany. In the end five schools with a total of 376 students (204 males and 172 females) participated in the study. All schools and students participated voluntarily and all participants’ and their parents’ consents were obtained. The students came from 16 different classes in grades 8 (*n* = 192) and 9 (*n* = 184). The average age of the students at the time of the assessment was *M* = 14.96 (*SD* = 0.91) years. According to the curriculum, all students were familiar with linear functions. The 8th graders had been introduced to the topic about 3 months before the study, the 9th graders had already worked on the topic in the previous school year. Accordingly, we expected that all students were familiar with all the tasks used in the survey. All the classes participating followed the same curriculum and used the same textbooks, according to their teachers.

### Materials

To assess self-efficacy in a task-specific way, we selected 20 items from a performance test on linear functions (Leuders et al., 2017). We discussed the selection of items in an expert interview with mathematics teachers and mathematics education researchers. We selected the items from a broad range of topics relating to linear functions. Furthermore, it was taken into account that the students should be familiar with the content of the tasks. The items were systematically selected in such a way that they varied with respect to the task characteristics of representation (graph/table), context (intra-mathematical/extra-mathematical) and operation (creating/reading), as described above. Each dimension was represented by ten items. A total of 14 of these items had been used in a pilot study (*N* = 120) which assessed students’ self-efficacy and performance. The other six items supplemented these 14 items to get a balanced mixed design. Each of the 20 single items has a distinctive feature in all three dimensions. Table 1 provides an overview of the number of tasks per dimension.

**TABLE 1:** Overview of the number of tasks per task dimension.

		Context	
Representation	Operation	Intra-mathematical	Extra-mathematical
Table	create	2	3
	read	3	2
Graph	create	2	2
	read	3	3

**FIGURE 2:**
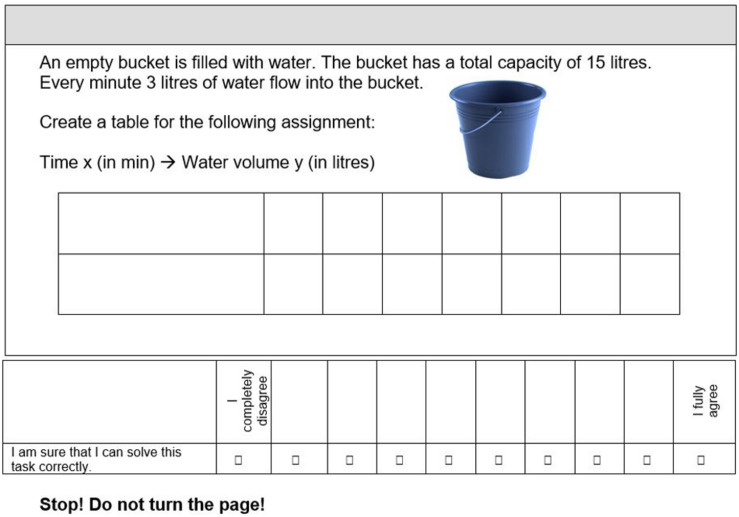
Sample Item.

Figure 2 shows a sample item. The item represents an extra-mathematical context, and studens have to create a table. The item in Figure 2 is extra-mathematical although it is only embedded in a context to a limited extent.

### Procedure

The assessment of self-efficacy took place in regular classrooms. Students received a booklet with 20 items. For each item, students were asked to look at the item for 30 s but not to solve it. They were then asked, without any time pressure, to indicate the degree of agreement with the statement “I am sure that I can solve this task correctly” on a ten-point Likert scale (from 1: “I completely disagree” to 10: “I fully agree”). The ten-point Likert scale was chosen in compliance with the procedure of other studies (Pajares et al., 2001; Pajares, 2003; Bandura, 2006). The time span of 30 s was used to prevent students from actually trying to solve the task. Prior pilot interviews suggested that a period of 30 s was suitable for this purpose. Overall, the assessment of self-efficacy took approximately 25 min. The assessment of self-efficacy was followed by a test session, in which students were asked to actually solve the same 20 tasks^1^.

### Data-Analysis

We used SPSS 25 (Arbuckle, 2013) for item analysis and also to provide descriptive statistics. We further used Mplus (Muthén and Muthén, 2017) to conduct confirmatory factor analysis (CFA) with the aim of understanding the theoretically assumed structure of self-efficacy. In this analysis, self-efficacy was modeled as a latent variable (Hu and Bentler, 2000; Kline, 2011). MacCallum (2000) suggests constructing a sequence of models ranging from those with a relatively simple structure (model 1: one latent variable, self-efficacy, underlying participants’ responses on all items, as well as models 2–4 with a between-item multi-dimensionality approach without a particular hierarchy) to those with a relatively complex structure (model 5: a within-item multi-dimensionality approach) (Aish and Jöreskog, 1990).

To determine the model fits, we tested for global and local fit values. The global fit values (also known as goodness of fit values) refer to the entire measurement model and distinguish between absolute Chi-square (*CMIN*), Root Mean Square Error of Approximation (*RMSEA*), Standard Root Mean Squared Residual (*SRMR*), incremental [estimate of comparative fit versus a null relation baseline model, named Comparative Fit Index (*CLI*) and Tucker Lewis Index (*TLI*)], and economy fit values (*CMIN/df*). A limitation of relying on the Chi-square statistics is that the model can be “adapted too closely to the sample at hand and [contain] too many parameters” (Arzheimer, 2016, p. 63). The goodness of fit values are more informative when the sample size increases (Kline, 2011). This is the reason why we mainly refer to the goodness of fit values.

Table 2 shows which values are acceptable and which are “good” according to Kline (2011). A good *RMSEA* value is lower than 0.05. It represents the proportion of information in the variance-covariance matrix that is not explained by the model. The *SRMR*-value is the square root of the average deviation of the model-predicted and empirical covariance-variance matrix. It should be lower than 0.05. The *TLI* and *CFI* values refer to the information proportions of the variance-covariance matrix compared to the independence model and should be greater than 0.95. The economy fit values *CMIN/df* should be lower than 1.5 and refer to the economy of a model (Kline, 2011).

**TABLE 2:** Global fit index according to Kline (2011).

	Absolute Fit					Incremental Fit		Economy	Comparison
	χ^2^	*p*	*df*	*RMSEA*	*SRMR*	*TLI*	*CFI*	*CMIN/df*	*BIC*
Acceptable Good		>0.05		<0.08 <0.05	<0.08 <0.05	>9 >0.95	>0.9 >0.95	<2 <1.5	smallest

*χ^2^, Chi-square; RMSEA, Root Mean Square Error of Approximation; SRMR, Standard Root Mean Squared Residual; TLI, Tucker-Lewis index; CFI, Comparative Fit Index; CMIN/df, criterion of economy; BIC, Bayesian Information Criterion.*

To test competing models, the model value of the *BIC* (Bayesian Information Criterion) can be used. Basically, the following applies: a model is considered better when the *BIC* decreases by about six points compared to another model (Raftery, 1995).

The absolute fit values are not always sufficient to judge whether the data adequately represent a theoretical model. For this reason, local fit values are also relevant; they can distinguish between convergent validity and discriminant validity. The convergent validity includes the indicator reliability, the average variance extracted (*AVE*), the *t*-value, and the factor reliability. The standard values are located together with the results in Table 7 (Bagozzi and Baumgartner, 1996). The discriminant validity is tested by means of the Fornell-Lacker criterion. It focuses on the correlation of two constructs and their separability (Fornell and Larcker, 1981). More precisely, on average it is empirically clarified that the variance of a construct is greater than the squared correlations of the construct with all other constructs considered (Kline, 2011).

## Results

### Descriptive Statistics

Overall, self-efficacy ratings were high for all 20 items (ranging from *M*_*min*_ = 5.22 to *M*_*max*_ = 8.90; on a scale from 1 to 10), suggesting that the participants were confident in their ability to solve most of the items correctly. Item-analysis of the distributions indicated that there was a left skewed distribution, which deviated significantly from a normal distribution in nearly all items. For this reason, further analyses were carried out with the Robust Maximum Likelihood estimation (Muthén and Muthén, 2017). Furthermore, item 1 (*P*_*i*_ = 0.83) and item 20 (*P*_*i*_ = 0.89) were excluded from further analyses due to high student ratings^2^ (Döring and Bortz, 2016). Table 3 shows the mean values, the standard deviation as well as the skewness and item-difficulty of all self-efficacy items^3^.

**TABLE 3:** Mean values (manifest), standard deviation (*SD*), item-difficulty (*P_*i*_)* and skewness.

	*M*	*SD*	*P*_*i*_	Skewness
Item1	8.26	2.33	0.82	−1.58
Item2	7.52	2.78	0.75	−0.93
Item3	8.23	2.46	0.82	−1.58
Item4	5.69	3.14	0.57	−0.08
Item5	6.31	3.25	0.63	−0.28
Item6	6.26	2.82	0.63	−0.31
Item7	5.22	2.97	0.52	0.17
Item8	7.70	2.61	0.77	−1.70
Item9	8.11	2.54	0.80	−1.32
Item10	6.39	3.23	0.40	−0.34
Item11	7.70	2.91	0.77	−1.09
Item12	7.21	3.06	0.72	−0.77
Item13	6.40	3.02	0.64	−0.38
Item14	7.21	2.80	0.72	−1.67
Item15	7.43	2.88	0.74	−0.91
Item16	7.14	2.70	0.71	−0.76
Item17	7.66	2.60	0.77	−1.03
Item18	7.22	2.65	0.72	−0.78
Item19	6.13	2.98	0.61	−0.33
Item20	8.90	2.23	0.89	−2.38

*The minimum of each item is 1, the maximum of each item is 10.*

### Confirmatory Factor Analysis

#### Global Fit Values

**FIGURE 3:**
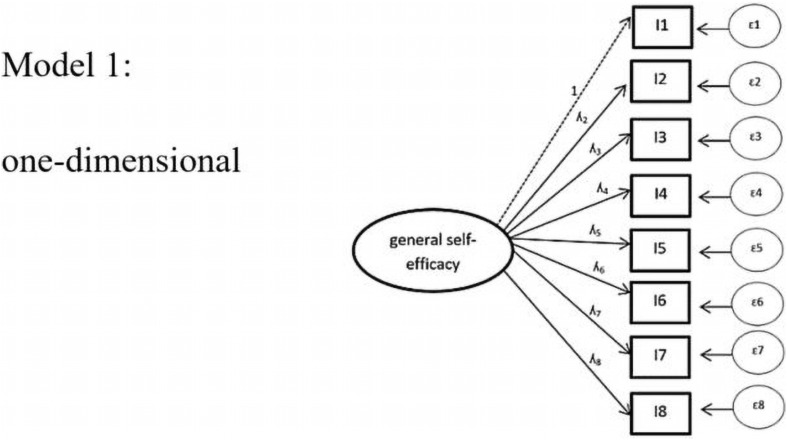
Model 1: one-dimensional. *ε* = error variances; *λ* = factor padding loading; *I* = item = task. For the sake of simplicity, the figure displays only 8 instead of all 18 items included in the analysis.

We first tested a one-dimensional model, which does not include task characteristics as factors (see Figure 3). As displayed in Table 4, the model exhibited acceptable values in all global fit values.

**TABLE 4:** Global fit values of the one-dimensional model.

	Absolute Fit					Incremental Fit		Economy	Comparison
	χ^2^	*p*	*df*	*RMSEA*	*SRMR*	*TLI*	*CFI*	*CMIN/df*	*BIC*
model 1: one-dimensional	254.16	0.001	135	0.048++	0.044++	0.93+	0.93+	1.89+	31208.25

*For thresholds of acceptable fit see Table 2. Good values are marked with ++. Acceptable values are marked with +.*

**FIGURE 4:**
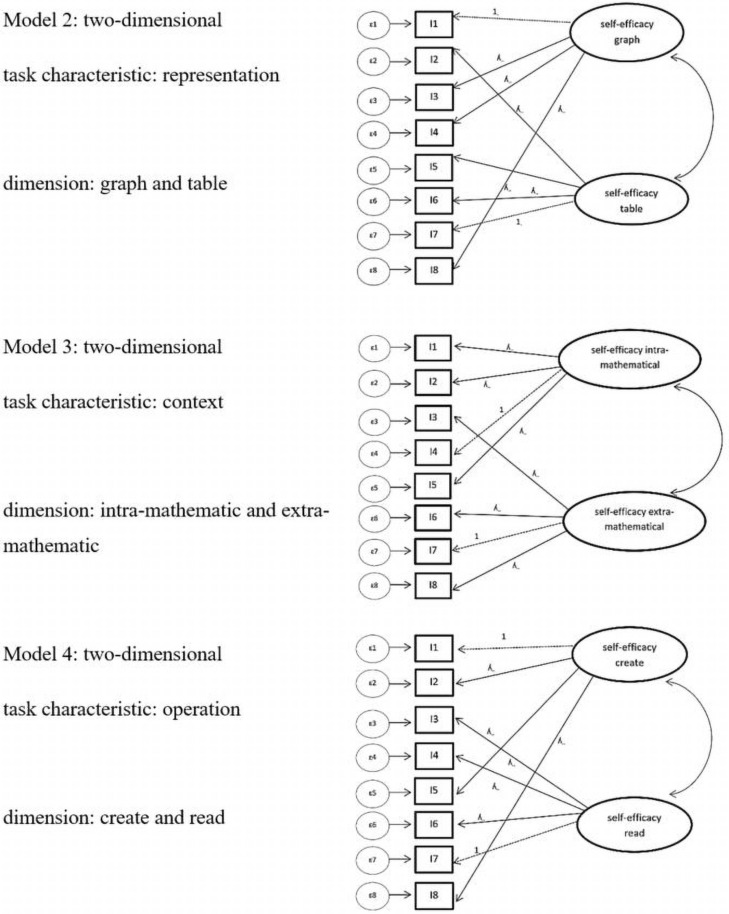
Model 2–4 task characteristic: Model of confirmatory factor analysis task characteristic differentiated in model 2: representation (graph or table), model 3: context (extra-mathematical or intra-mathematical), and model 4: operation (create and read). For simplification, only 8 items are shown. *ε* = error variances; *λ* = factor padding loading; *I* = item = task.

Next, we tested two-dimensional models, which each include the two dimensions of representation (table/graph; model 2), the context (intra-mathematical/extra-mathematical; model 3), or the operation (create/read; model 4) (see Figure 4).

As Table 5 displays, model 3 shows good values in the different global fits. In contrast, model 2 and model 4 only show acceptable global fit values, with better values for model 2 than model 4. The *BIC* values (lower is better) indicate that all three models had better fit values than the one-dimensional model 1, and that among the three two-dimensional models, model 3 had the best global fit values. Furthermore, the *BIC* values indicate that in direct comparison of models with acceptable fit indices, model 3 fits the data better because the *BIC* difference between model 3 and model 2 is lower by the value 43 and the *BIC* difference between model 3 and model 4 is lower by the value of 51. Furthermore, the likelihood-ratio test (Kline, 2011) showed significantly better results for model 3 (*χ^2^*(1) = 42.67, *p* < 0.001) than model 1, as well as for model 2 than for model 1 (*χ^2^*(1) = 10.73 *p* < 0.001).

**TABLE 5:** Global fit values of models 2–4 (each two-dimensional) task characteristic.

	Absolute Fit					Incremental Fit		Economy	Comparison
	*CMIN*	*p*	*df*	*RMSEA*	*SRMR*	*TLI*	*CFI*	*CMIN/df*	*BIC*
model 2: representation	243.43	0.001	134	0.047++	0.043++	0.93+	0.94+	1.82+	31199.22
model 3: context	211.49	0.001	134	0.039++	0.040++	0.95++	0.96++	1.57+	31156.10++
model 4: operation	251.41	0.001	134	0.048++	0.044++	0.93+	0.94+	1.90+	31207.11

*For thresholds of acceptable fit see Table 2. Good values are marked with ++. Acceptable values are marked with +.*

**FIGURE 5:**
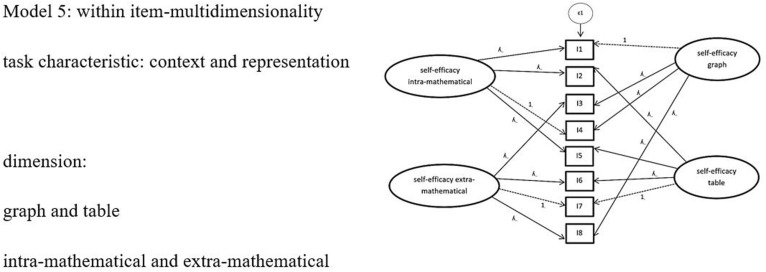
Complex Model 5: Model of confirmatory factor analysis with two task characteristics context and representation. For simplification, only 8 items and one epsilon are shown. *ε* = error variances; *λ* = factor padding loading; *I* = item = task.

Considering the results of models 2–4, which were all between-item multi-dimensionality models, we tested one more complex within-item multi-dimensionality model (model 5). Because models 2 and 3 were the two models with the best global fit among the two-dimensional models, and had better *BIC* values [differences higher than 6 according to Raftery (1995)] than the one-dimensional model, we included the dimensions of the task characteristics of representation and context in model 5 (see Figure 5).

The results (see Table 6) showed that this model did not have acceptable fit values.

**TABLE 6:** Global fit values model 5 with task characteristic representation and context.

	Absolute Fit					Incremental Fit		Economy	Comparison
	*CMIN*	*p*	*df*	*RMSEA*	*SRMR*	*TLI*	*CFI*	*CMIN/df*	*BIC*
model 5 representation and context	480.94	0.001	117	0.09	0.24	0.75	0.81	4.11	31573.01

*For thresholds of acceptable fit see Table 2.*

Finally, for the purpose of comparison, we created a two-dimensional random model in which all items were assigned randomly to one of the two dimensions. The random model showed no better results than models 1–4 (χ^2^(134) = 251.44 *p* < 0.001; *RMSEA* = 0.048; *SRMR* = 0.044; *TLI* = 0.929; *CFI* = 0.938 *BIC* = 31209.97).

In conclusion, the one-dimensional model 1 along with model 2 (representation) and model 4 (operation) only exhibit acceptable values. The more complex model 5, on the other hand, did not have acceptable fit values. Model 3 (context) showed the best global fit values.

#### Local Fit Values

We further analyzed the local fit values for those models which had acceptable global fit values (i.e., models 1–4). Table 7 shows that all models have good *t*-values, factor reliability and *AVE*. However, the indicator-reliabilities are not acceptable for all models. Model 1 (one-dimensional) and model 2 (representation) have items which are outside the acceptable range of 0.3. These items have a low share of variance for the factor and should be excluded from the model. For model 3 (context) and model 4 (operation), all items have the acceptable value of over 0.3.

**TABLE 7:** Local fit values.

	Indicator variable	Indicator reliability	t- value	Factor reliability	AVE	r	Fornell-Lacker
threshold		>0.3	>2	>0.6	>0.5		
model 1: one-dimensional		0.24–0.49	6.47–11.21***	0.99	0.83		
model 2: representation	graph	0.24–0.50	5.56–11.40***	0.98	0.84	0.91	fulfilled
	table			0.98	0.83		fulfilled
model 3: context	intra	0.32–0.53	6.60–11.68***	0.98	0.83	0.80	fulfilled
	extra			0.98	0.85		fulfilled
model 4: operation	create	0.30–0.50	6.48–11.25***	0.98	0.84	0.96	not fulfilled
	read			0.97	0.82		not fulfilled

For thresholds of acceptable fit see Bagozzi and Baumgartner (1996) and Hair (1995). For the Fornell-Lacker criterion see Fornell and Larcker (1981). ***p < 0.001.

The correlation (r) between the respective latent constructs varies depending on the model. There was a strong correlation between the different latent variables ranging from *r* = 0.80 (model 3: context) to *r* = 0.91 (model 2: representation) to 0.96 (model 4: operation). At first glance, the high correlation between the respective latent constructs seems alarming. The high correlation raises the question whether the two dimensions are actually separable. The Fornell-Lacker criterion, which focused on the correlation as compared to the *AVE*, is not fulfilled in all models. Only for model 2 (representation) and model 3 (context) was the criterion fulfilled. Hence, it appears that a separation of the dimensions (e.g., for context the separation of intra-mathematical from extra-mathematical) is possible.

In conclusion, the results of the local fit values confirm the results of the global fit values. Model 3 (context) seems to be the best model for all values.

Finally, we briefly report the reliabilities for the dimensions in models 2 and 3. A reliability analysis using Cronbach’s alpha showed *Cronbach’s alpha* = 0.87 for the intra-mathematical items, *Cronbach’s alpha* = 0.86 for the extra-mathematical items, *Cronbach’s alpha* = 0.83 for the items with a graph and *Cronbach’s alpha* = 0.82 for the items with a table.

## Discussion

The aim of this study was to explore which task characteristics are relevant when students evaluate their own ability to perform mathematical tasks with linear functions successfully. Bandura (1997) clearly stated that when developing self-efficacy scales, researchers should draw on conceptual analysis and the knowledge of experts to find out what it takes to succeed in a given pursuit. The study draws its data both from a conceptual analysis and the expert knowledge from mathematics educators and learning experts, to construct a self-efficacy scale that takes account of the salient aspects of solving linear functions. The study advances previous research by including multiple task characteristics which have not been considered in combination yet. A distinction was made between three task characteristics, namely those of the representational form (graph and table), the context (intra-mathematical and extra-mathematical), and the operation (create and read). We expected that these three task characteristics potentially affect students’ self-efficacy because all students have gained experience with tasks in these formats. All of these task characteristics should be familiar for students.

### Is Self-Efficacy a One-Dimensional Construct or Is It a Multi-Dimensional Construct Along the Dimensions of the Task Characteristic?

Previous research (e.g., Chen and Zimmerman, 2007; Bonne and Johnston, 2016) assumed one-dimensional models of self-efficacy or a multi-dimensionality of self-efficacy (e.g., Bruning et al., 2013; Street et al., 2017) without focusing on concrete tasks or task characteristics. With such a premise, it is not necessary to consider specific task characteristics because students are assumed to relate a presented task to the area of self-efficacy in question. However, in our study the two-dimensional models, which assume that students do in fact assess their self-efficacy differently depending on task characteristics, fit our data better than a one-dimensional model. More specifically, students appear to differentiate in their self-efficacy between tasks with and without context as well as between tasks with different representational forms. As examined in previous studies such as Acevedo Nistal et al. (2013), students who are given a choice of a representational form are clearly influenced by both the subject and choice of the task itself. The subject-related justification could be explained by differences in self-efficacy for different task characteristics. However, the study here provides support that students do not rely on all relevant characteristics (representation, context, and operation) of a task when they evaluate their own abilities. The results underline the complexity of task characteristics in the context of linear functions (Leinhardt et al., 1990).

### The Task Characteristics of the Context Represented the Data Best

Model 3 (context) showed the best values at both global and local levels. This can be explained in two ways. Firstly, students may have had learning experiences with the strongest influence on their self-efficacy when tasks contained a context. This would be in line with an often-reported dislike of word-problems (Van Dooren et al., 2018). Secondly, it is also possible that during assessment the task characteristics relating to context were the most salient, so that when evaluating their abilities students tended to perceive these characteristics more easily, whether intra- or extra-mathematical. The results go hand in hand with the important role of context in mathematical situations, as stated above (Bock et al., 2015; Van Dooren et al., 2018). Our results seem to differ from those of Schukajlow et al. (2012) who also assessed self-efficacy in a task-specific manner. In their study, Schukajlow et al. formed three item groups for self-efficacy in modeling problems, intra-mathematical tasks and word problems. In each group they used tasks on linear functions as well as on Pythagoras’s theorem. They found no difference in the mean values of the self-efficacies defined by these three groups of tasks. However, the authors did not perform an analysis of the dimensionality and therefore did not make a statement about whether self-efficacy in their definition was to be considered a one-dimensional or multi-dimensional construct.

Similarly, model 2 (representation) had better global fit values than the one-dimensional model, although some items had to be excluded, and in direct comparison the model 2 had a worse fit than model 3. This emphasizes the fact that representation plays an important role in students’ self-evaluation, and this goes along with the results of research in the role of representational forms for performance (Keller and Hirsch, 1998; Duval, 2006). In direct comparison (*BIC*) to model 3 (context), model 2 (representation) indicated a worse fit.

Model 4 (operation) had no better *BIC* values than the one-dimensional model. At the local level it did not seem possible to separate the creating and reading dimensions. This suggests that the operation did not play a similarly important role in students’ self-evaluation as the previous models. Again, two explanations are possible. First, students have not experienced the operation as relevant affordance in tasks during their learning history. Second, it may also be the case that students do not spontaneously perceive the importance of the operation which is required to solve the task. Since research has shown the role of operations in performance situations (Rolfes et al., 2018), one may assume that salience might be a better explanation for our results.

The adequacy of the two-dimensional models 2 and 3, each focusing on one task characteristic, encouraged further analyses in a within-item multi-dimensionality approach. The main assumption of model 5 (representation/context) was that students rely on the context while also taking the representational form into account and then came to conclusions about their abilities. However, the analysis of model 5 showed that there were no acceptable global fit values. This may have been caused by a focusing mechanism: learners do not simultaneously rely on the representational form and the context of a task while evaluating their abilities, but rather rely on only one aspect, i.e., the context in which a mathematical task is embedded. Additionally, the economy fit value *CMIN/df* of 4.1 was relatively high. It is possible that with an even larger sample, a higher number of degrees of freedom would lead to a better fit value for such a complex model.

In conclusion, the findings with respect to a task-specific assessment of self-efficacy confirm the theoretical assumption that self-efficacy is not a one-dimensional construct. A comparison of the two best models (“representation” and “context”) showed that the context model is a significantly better model.

The results of this study are relevant for future research on self-efficacy with task-specific assessments in at least three ways. First, according to the findings by Marsh et al. (2018) or Pajares and Kranzler (1995), the results suggest that it is very important to use a task-specific assessment because the construct of self-efficacy is inherently dependent not only on the domain but also on the task type. Second, it is important to select tasks carefully and to analyze the required abilities. Third, the mathematical educational perspective showed that subject-related justifications (Acevedo Nistal et al., 2013) on tasks could be explained by self-efficacy.

### Limitations

Our study has at least four limitations. First, the sample consisted of *N* = 376 students of similar age and with a very similar curricular background. It is possible that a variation in cognitive and curricular conditions across, for example, different school types would produce different results. In a similar manner, it could be possible that model 2 (representation) and model 4 (operation) could show a better fit, due to the students’ different learning trajectories. Moreover, the most complex model (model 5) would perhaps show a better fit with an even larger sample (higher value of *df*).

Second, the study showed that among the models we tested, some fit better than others. Of course, we were not able to test all possible models. Accordingly, we cannot rule out the possibility that even more complex models, or models that include other task characteristics not considered here, fit the data even better. However, we do not think this is very likely because we derived our models from careful theoretical analyses of the content domain (linear functions) and previous empirical findings.

Third, a further limitation may result from our focus on linear functions. It is possible that an assessment in other areas of mathematics would lead to different results. In particular, it remains an open question whether the context of the tasks would also be the most salient task characteristic in a different content area, or whether other characteristics, such as the representational form, would be more salient. One can assume that in areas such as binomial formulae, where extra-mathematical contexts do not typically play an important role, students would rely on other task characteristics to assess their own abilities.

Fourth, it should also be considered that self-efficacy was recorded with the help of a printed booklet. Although students received explicit instructions when to turn pages, it was not possible to ensure that all students actually followed these instructions (e.g., turning pages after 30 s). One way to avoid this issue would be to present problems to the whole class using a projector, or by a computer-based assessment. These assessments would, however, reduce the validity of the assessment, since students commonly solve mathematical problems on paper.

### Further Research and Implications

The present study focused on the assessment of students’ self-efficacy, although we also assessed students’ performance on the same tasks. While we identified a multi-dimensional structure in self-efficacy, similar *CFA* analyses for the performance test suggested a one-dimensional structure. This result requires further investigation, particularly because an earlier study of students’ performance with similar items did detect a multi-dimensional structure of performance as well (Leuders et al., 2017). More generally, further research is needed to better understand the relation between students’ task-specific self-efficacy and their performance on the very same tasks, and the factors that influence this relation (Siefer et al., 2020).

Another issue for further research is in how far the results can be generalized to include other contexts. A worthwhile next step might be to extend the dimensionality analysis to other mathematical domains (e.g., geometry) or to other special topics (e.g., Pythagoras’s theorem). It would then be interesting to see whether it is possible to identify overarching task characteristics (e.g., a real-life context) that are relevant for students’ self-efficacy in all topics.

Within a task-specific assessment, it could be interesting to run validation studies to compare the two forms of task-specific assessments (indirect vs. direct assessment, described above). One can expect that there will not (necessarily) be a high correlation between the different assessments because of the higher abstraction of the different forms. For example, when students respond to the question “I can work with graphs,” they may think of a wide variety of operations in dealing with graphs, while in a task-specific assessment, the concrete operation is presented in the given task. To understand students’ thinking better during their assessment of self-efficacy, one could use qualitative methods. For example, one could ask question such as: “What features of the task have you considered?” A limitation is that such a question could stimulate students to reflect on the tasks, which could lead to a biased measurement of subsequent tasks. Another less invasive method could be eye-tracking, which could provide insights into perception processes (Holmqvist, 2015; Nugteren et al., 2018).

The results of this study may be used to support student learning in different ways. A reflected assessment of one’s own abilities in which all task characteristics can be taken into account may result in higher accuracy (Chen, 2003). The results underline the fact that in spontaneous evaluation processes of abilities, students focus particularly on the context and the representational form of the tasks. Different prompts could encourage students to consider other task characteristics as well, which could result in higher accuracy of the assessment, in relation to actual performance (Chen, 2003).

### Conclusion

This study emphasized the importance of task characteristics in the assessment of students’ self-efficacy. Self-efficacy appears to be a multi-dimensional construct even within a specific mathematical topic. The study showed the empirical separability of self-efficacy dimensions related to linear functions according to task characteristics. Future research should consider more strongly the specific demands of a domain when assessing students’ self-efficacy. On a more general note, the study showed the importance of the specificity of the domain and subject-matter when assessing a psychological construct.

## Data Availability Statement

The raw data supporting the conclusions of this article will be made available by the authors, without undue reservation.

## Ethics Statement

The studies involving human participants were reviewed and approved by the Ministry of Culture, Youth and Sports Baden-Württemberg. Written informed consent to participate in this study was provided by the participants’ legal guardian/next of kin.

## Author Contributions

KS collected and the data. KS, TL, and AO interpreted the data and wrote the manuscript. All authors developed the concept of the study, regular exchange about the article, and contributed equally to its success.

## Conflict of Interest

The authors declare that the research was conducted in the absence of any commercial or financial relationships that could be construed as a potential conflict of interest.

## Footnotes

1 For the purpose of the present article, we did not systematically analyze the performance data. However, in a preliminary analyses, a confirmatory factor analysis suggested that a confirmatory performance was a one-dimensional construct χ^2^(170) = 240.40 *p* < 0.001; *RMSEA* = 0.033; *SRMR* = 0.044; *TLI* = 0.902; *CFI* = 0.912), that is, students did not differ in their performance due to task characteristics. The one-dimensional model was tested against the same two-dimensional models as for self-efficacy. The *BIC* values were always found to be better for the one-dimensional model. There were no significant differences in the linear functions test between the 8 and 9 graders *t*(374) = 0.979; *p* = 0.328. Hence, merging the classes seemed to have no further effect on the results.

2 The higher the value is (max 1), the more students have responded that they are confident about being able to solve this task correctly. So the item was too easy.

3 A comparison of self-efficacy for grade levels showed significant differences between 8th and 9th graders (*t*(359.15) = –2.33; *p* = 0.02). 9th graders showed slightly higher self-efficacy (*M* = 7.35; *SD* = 1.5) than 8th graders (*M* = 6.93; *SD* = 1.9). However, the effect size of this difference was fairly small (*d* = 0.24). Because there were no theoretical reasons to assume grade level differences in the dimensionality of self-efficacy, and in order not to reduce statistical power for our model analyses, we did not include grade level as a factor in our models.
