# Supplementary material for: Which Task Characteristics Do Students Rely on When They Evaluate Their Abilities to Solve Linear Function Tasks? – A Task-Specific Assessment of Self-Efficacy
Source: Front Psychol. 2021 Mar 12;12:596901. doi: 10.3389/fpsyg.2021.596901 (PMC7994344; doi:10.3389/fpsyg.2021.596901)
Supplement: Supplementary file 1 [file Table_1.pdf]

Supplementary Material

Mplus VERSION 8.3

MUTHEN & MUTHEN

02/09/2020 12:07 PM

INPUT INSTRUCTIONS

Title: CFA mit Dimension Repräsentation zur Überprüfung generisch

Data: File is Daten_aufbereitet_CFA.dat;

Variable: Names are SWE1-SWE20 msk1-msk7;

usevariable = SWE2- SWE19;

MODEL:

generisch BY SWE2 SWE3 SWE4 SWE5 SWE5 SWE6 SWE7 SWE8 SWE9 SWE10 SWE11

SWE12 SWE13 SWE14 SWE15 SWE16 SWE17 SWE18 SWE19;

Analysis: type=general;

Estimator is MLR;

Output: sampstat STDYX; modindices;

INPUT READING TERMINATED NORMALLY

CFA mit Dimension Repräsentation

SUMMARY OF ANALYSIS

Number of groups 1

Number of observations 376

Number of dependent variables 18

Number of independent variables 0

Number of continuous latent variables 1

Observed dependent variables

Continuous

SWE2 SWE3 SWE4 SWE5 SWE6 SWE7

SWE8 SWE9 SWE10 SWE11 SWE12 SWE13

SWE14 SWE15 SWE16 SWE17 SWE18 SWE19

Continuous latent variables

GENERISCH

Estimator MLR

Information matrix OBSERVED

Maximum number of iterations 1000

Convergence criterion 0.500D-04

Maximum number of steepest descent iterations 20

Input data file(s)

Daten_aufbereitet_CFA.dat

Input data format FREE

SAMPLE STATISTICS

SAMPLE STATISTICS

Means

SWE2	SWE3	SWE4	SWE5	SWE6
7.519	8.231	5.689	6.311	6.255

Means

SWE7	SWE8	SWE9	SWE10	SWE11
5.218	7.702	8.106	6.388	7.697

Means

SWE12	SWE13	SWE14	SWE15	SWE16
7.207	6.404	8.258	7.428	7.144

Means

SWE17	SWE18	SWE19
7.662	7.221	6.133

Covariances

	SWE2	SWE3	SWE4	SWE5	SWE6
SWE2	7.686				
SWE3	2.359	6.040			
SWE4	2.496	2.245	9.821		
SWE5	3.264	2.877	3.397	10.523	
SWE6	2.578	3.066	3.335	3.944	7.956
SWE7	2.844	2.460	3.767	3.570	3.990
SWE8	2.059	2.838	2.418	2.579	3.081
SWE9	2.360	2.688	2.374	3.025	2.861

SWE10	2.091	3.038	3.453	4.233	3.369
SWE11	2.875	3.150	2.770	3.557	3.056
SWE12	4.081	2.194	2.841	2.986	3.032
SWE13	2.695	2.561	3.493	3.332	3.136
SWE14	1.970	2.387	1.929	2.656	2.511
SWE15	2.544	2.361	2.742	3.383	3.016
SWE16	2.335	3.315	1.872	2.466	3.314
SWE17	2.808	3.488	2.060	2.254	2.698
SWE18	2.965	2.510	2.279	2.594	3.906
SWE19	2.963	2.828	3.036	3.381	3.700

Covariances

	SWE7	SWE8	SWE9	SWE10	SWE11
SWE7	8.798				
SWE8	2.887	6.778			
SWE9	2.721	2.800	6.451		
SWE10	3.753	2.525	2.799	10.392	
SWE11	3.821	2.958	3.205	3.035	8.440
SWE12	2.846	2.509	3.507	2.640	2.877
SWE13	3.274	2.317	2.728	3.593	4.011
SWE14	1.901	2.739	2.659	2.254	2.762
SWE15	2.837	2.859	3.359	3.206	3.287
SWE16	2.860	3.200	2.559	3.354	2.903
SWE17	2.813	3.091	2.626	2.663	3.233
SWE18	3.433	3.414	2.695	2.574	3.301

SWE19	3.351	3.399	2.731	2.999	3.676

Covariances

	SWE12	SWE13	SWE14	SWE15	SWE16
SWE12	9.319				
SWE13	2.961	9.076			
SWE14	2.827	2.874	5.404		
SWE15	3.595	3.524	2.940	8.282	
SWE16	2.390	2.955	3.048	2.577	7.277
SWE17	2.751	3.113	2.845	3.131	3.751
SWE18	3.106	2.804	2.791	3.328	3.867
SWE19	3.153	3.473	3.149	3.680	3.598

Covariances

	SWE17	SWE18	SWE19
SWE17	6.740		
SWE18	3.436	7.023	
SWE19	3.266	4.189	8.849

Correlations

	SWE2	SWE3	SWE4	SWE5	SWE6
SWE2	1.000				
SWE3	0.346	1.000			
SWE4	0.287	0.291	1.000		
SWE5	0.363	0.361	0.334	1.000	
SWE6	0.330	0.442	0.377	0.431	1.000

SWE7	0.346	0.337	0.405	0.371	0.477
SWE8	0.285	0.443	0.296	0.305	0.420
SWE9	0.335	0.431	0.298	0.367	0.399
SWE10	0.234	0.383	0.342	0.405	0.371
SWE11	0.357	0.441	0.304	0.377	0.373
SWE12	0.482	0.292	0.297	0.302	0.352
SWE13	0.323	0.346	0.370	0.341	0.369
SWE14	0.306	0.418	0.265	0.352	0.383
SWE15	0.319	0.334	0.304	0.362	0.372
SWE16	0.312	0.500	0.221	0.282	0.436
SWE17	0.390	0.547	0.253	0.268	0.368
SWE18	0.404	0.385	0.274	0.302	0.523
SWE19	0.359	0.387	0.326	0.350	0.441

Correlations

	SWE7	SWE8	SWE9	SWE10	SWE11
SWE7	1.000				
SWE8	0.374	1.000			
SWE9	0.361	0.423	1.000		
SWE10	0.393	0.301	0.342	1.000	
SWE11	0.443	0.391	0.434	0.324	1.000
SWE12	0.314	0.316	0.452	0.268	0.324
SWE13	0.366	0.295	0.357	0.370	0.458
SWE14	0.276	0.453	0.450	0.301	0.409
SWE15	0.332	0.382	0.459	0.346	0.393

SWE16	0.357	0.456	0.373	0.386	0.370
SWE17	0.365	0.457	0.398	0.318	0.429
SWE18	0.437	0.495	0.400	0.301	0.429
SWE19	0.380	0.439	0.361	0.313	0.425

Correlations

	SWE12	SWE13	SWE14	SWE15	SWE16
SWE12	1.000				
SWE13	0.322	1.000			
SWE14	0.398	0.410	1.000		
SWE15	0.409	0.406	0.439	1.000	
SWE16	0.290	0.364	0.486	0.332	1.000
SWE17	0.347	0.398	0.471	0.419	0.536
SWE18	0.384	0.351	0.453	0.436	0.541
SWE19	0.347	0.388	0.455	0.430	0.448

Correlations

	SWE17	SWE18	SWE19
SWE17	1.000		
SWE18	0.499	1.000	
SWE19	0.423	0.531	1.000

Model 2: Representation

Mplus VERSION 8.3

MUTHEN & MUTHEN

02/09/2020 12:03 PM

INPUT INSTRUCTIONS

```
Title: CFA mit Dimension Repräsentation zur Überprüfung von Amos
Data: File is Daten_aufbereitet_CFA.dat;
Variable: Names are SWE1-SWE20;
usevariable SWE2-SWE19;
MODEL: Graph by SWE2 SWE5 SWE6 SWE8 SWE9 SWE12 SWE14 SWE15 SWE18 SWE19;
       Tabelle by SWE3 SWE4 SWE7 SWE10 SWE11 SWE13 SWE16 SWE17;
       Tabelle with Graph;
Analysis: type=general;
Estimator is MLR;
Output: sampstat standardized STDYX; modindices;
```

INPUT READING TERMINATED NORMALLY

SUMMARY OF ANALYSIS

Number of groups	1
Number of observations	376
Number of dependent variables	18
Number of independent variables	0
Number of continuous latent variables	2

Observed dependent variables

Continuous

SWE2	SWE3	SWE4	SWE5	SWE6	SWE7
SWE8	SWE9	SWE10	SWE11	SWE12	SWE13

SWE14 SWE15 SWE16 SWE17 SWE18 SWE19

Continuous latent variables

GRAPH TABELLE

Estimator MLR

Information matrix OBSERVED

Maximum number of iterations 1000

Convergence criterion 0.500D-04

Maximum number of steepest descent iterations 20

Input data file(s)

Daten_aufbereitet_CFA.dat

Input data format FREE

SAMPLE STATISTICS

SAMPLE STATISTICS

Means

SWE2	SWE3	SWE4	SWE5	SWE6
7.519	8.231	5.689	6.311	6.255

Means

SWE7	SWE8	SWE9	SWE10	SWE11
5.218	7.702	8.106	6.388	7.697

Means

SWE12	SWE13	SWE14	SWE15	SWE16
7.207	6.404	8.258	7.428	7.144

Means

SWE17	SWE18	SWE19
7.662	7.221	6.133

Covariances

	SWE2	SWE3	SWE4	SWE5	SWE6
SWE2	7.686				
SWE3	2.359	6.040			
SWE4	2.496	2.245	9.821		
SWE5	3.264	2.877	3.397	10.523	
SWE6	2.578	3.066	3.335	3.944	7.956
SWE7	2.844	2.460	3.767	3.570	3.990
SWE8	2.059	2.838	2.418	2.579	3.081
SWE9	2.360	2.688	2.374	3.025	2.861
SWE10	2.091	3.038	3.453	4.233	3.369
SWE11	2.875	3.150	2.770	3.557	3.056
SWE12	4.081	2.194	2.841	2.986	3.032
SWE13	2.695	2.561	3.493	3.332	3.136
SWE14	1.970	2.387	1.929	2.656	2.511
SWE15	2.544	2.361	2.742	3.383	3.016
SWE16	2.335	3.315	1.872	2.466	3.314
SWE17	2.808	3.488	2.060	2.254	2.698
SWE18	2.965	2.510	2.279	2.594	3.906

SWE19	2.963	2.828	3.036	3.381	3.700

Covariances

	SWE7	SWE8	SWE9	SWE10	SWE11
SWE7	8.798				
SWE8	2.887	6.778			
SWE9	2.721	2.800	6.451		
SWE10	3.753	2.525	2.799	10.392	
SWE11	3.821	2.958	3.205	3.035	8.440
SWE12	2.846	2.509	3.507	2.640	2.877
SWE13	3.274	2.317	2.728	3.593	4.011
SWE14	1.901	2.739	2.659	2.254	2.762
SWE15	2.837	2.859	3.359	3.206	3.287
SWE16	2.860	3.200	2.559	3.354	2.903
SWE17	2.813	3.091	2.626	2.663	3.233
SWE18	3.433	3.414	2.695	2.574	3.301
SWE19	3.351	3.399	2.731	2.999	3.676

Covariances

	SWE12	SWE13	SWE14	SWE15	SWE16
SWE12	9.319				
SWE13	2.961	9.076			
SWE14	2.827	2.874	5.404		
SWE15	3.595	3.524	2.940	8.282	
SWE16	2.390	2.955	3.048	2.577	7.277
SWE17	2.751	3.113	2.845	3.131	3.751

SWE18	3.106	2.804	2.791	3.328	3.867
SWE19	3.153	3.473	3.149	3.680	3.598

Covariances

	SWE17	SWE18	SWE19
SWE17	6.740		
SWE18	3.436	7.023	
SWE19	3.266	4.189	8.849

Correlations

	SWE2	SWE3	SWE4	SWE5	SWE6
SWE2	1.000				
SWE3	0.346	1.000			
SWE4	0.287	0.291	1.000		
SWE5	0.363	0.361	0.334	1.000	
SWE6	0.330	0.442	0.377	0.431	1.000
SWE7	0.346	0.337	0.405	0.371	0.477
SWE8	0.285	0.443	0.296	0.305	0.420
SWE9	0.335	0.431	0.298	0.367	0.399
SWE10	0.234	0.383	0.342	0.405	0.371
SWE11	0.357	0.441	0.304	0.377	0.373
SWE12	0.482	0.292	0.297	0.302	0.352
SWE13	0.323	0.346	0.370	0.341	0.369
SWE14	0.306	0.418	0.265	0.352	0.383
SWE15	0.319	0.334	0.304	0.362	0.372

SWE16	0.312	0.500	0.221	0.282	0.436
SWE17	0.390	0.547	0.253	0.268	0.368
SWE18	0.404	0.385	0.274	0.302	0.523
SWE19	0.359	0.387	0.326	0.350	0.441

Correlations

	SWE7	SWE8	SWE9	SWE10	SWE11
SWE7	1.000				
SWE8	0.374	1.000			
SWE9	0.361	0.423	1.000		
SWE10	0.393	0.301	0.342	1.000	
SWE11	0.443	0.391	0.434	0.324	1.000
SWE12	0.314	0.316	0.452	0.268	0.324
SWE13	0.366	0.295	0.357	0.370	0.458
SWE14	0.276	0.453	0.450	0.301	0.409
SWE15	0.332	0.382	0.459	0.346	0.393
SWE16	0.357	0.456	0.373	0.386	0.370
SWE17	0.365	0.457	0.398	0.318	0.429
SWE18	0.437	0.495	0.400	0.301	0.429
SWE19	0.380	0.439	0.361	0.313	0.425

Correlations

	SWE12	SWE13	SWE14	SWE15	SWE16
SWE12	1.000				
SWE13	0.322	1.000			
SWE14	0.398	0.410	1.000		

SWE15	0.409	0.406	0.439	1.000	
SWE16	0.290	0.364	0.486	0.332	1.000
SWE17	0.347	0.398	0.471	0.419	0.536
SWE18	0.384	0.351	0.453	0.436	0.541
SWE19	0.347	0.388	0.455	0.430	0.448

Correlations

	SWE17	SWE18	SWE19
SWE17	1.000		
SWE18	0.499	1.000	
SWE19	0.423	0.531	1.000

Model 3: Context

Mplus VERSION 8.3

MUTHEN & MUTHEN

02/09/2020 12:05 PM

INPUT INSTRUCTIONS

```
Title: CFA mit Dimension Repräsentation zur Überprüfung von Kontext mit robust
Data: File is Daten_aufbereitet_CFA.dat;
Variable: Names are SWE1-SWE20;
usevariable SWE2-SWE19;
MODEL:
    innermath by SWE4 SWE2 SWE5 SWE7 SWE9 SWE10 SWE11 SWE12 SWE13 SWE15;
    ausermath by SWE6 SWE3 SWE8 SWE14 SWE16 SWE17 SWE18 SWE19;
```

Analysis: type=general;

Estimator is MLR;

Output: sampstat standardized STDYX; modindices;

INPUT READING TERMINATED NORMALLY

SUMMARY OF ANALYSIS

Number of groups 1

Number of observations 376

Number of dependent variables 18

Number of independent variables 0

Number of continuous latent variables 2

Observed dependent variables

Continuous

SWE2 SWE3 SWE4 SWE5 SWE6 SWE7

SWE8 SWE9 SWE10 SWE11 SWE12 SWE13

SWE14 SWE15 SWE16 SWE17 SWE18 SWE19

Continuous latent variables

INNERMAT AUSERMAT

Estimator MLR

Information matrix OBSERVED

Maximum number of iterations 1000

Convergence criterion 0.500D-04

Maximum number of steepest descent iterations 20

Input data file(s)

Daten_aufbereitet_CFA.dat

Input data format FREE

SAMPLE STATISTICS

SAMPLE STATISTICS

Means

SWE2	SWE3	SWE4	SWE5	SWE6
7.519	8.231	5.689	6.311	6.255

Means

SWE7	SWE8	SWE9	SWE10	SWE11
5.218	7.702	8.106	6.388	7.697

Means

SWE12	SWE13	SWE14	SWE15	SWE16
7.207	6.404	8.258	7.428	7.144

Means

SWE17	SWE18	SWE19
7.662	7.221	6.133

Covariances

	SWE2	SWE3	SWE4	SWE5	SWE6
SWE2	7.686				
SWE3	2.359	6.040			
SWE4	2.496	2.245	9.821		

SWE5	3.264	2.877	3.397	10.523	
SWE6	2.578	3.066	3.335	3.944	7.956
SWE7	2.844	2.460	3.767	3.570	3.990
SWE8	2.059	2.838	2.418	2.579	3.081
SWE9	2.360	2.688	2.374	3.025	2.861
SWE10	2.091	3.038	3.453	4.233	3.369
SWE11	2.875	3.150	2.770	3.557	3.056
SWE12	4.081	2.194	2.841	2.986	3.032
SWE13	2.695	2.561	3.493	3.332	3.136
SWE14	1.970	2.387	1.929	2.656	2.511
SWE15	2.544	2.361	2.742	3.383	3.016
SWE16	2.335	3.315	1.872	2.466	3.314
SWE17	2.808	3.488	2.060	2.254	2.698
SWE18	2.965	2.510	2.279	2.594	3.906
SWE19	2.963	2.828	3.036	3.381	3.700

Covariances

	SWE7	SWE8	SWE9	SWE10	SWE11
SWE7	8.798				
SWE8	2.887	6.778			
SWE9	2.721	2.800	6.451		
SWE10	3.753	2.525	2.799	10.392	
SWE11	3.821	2.958	3.205	3.035	8.440
SWE12	2.846	2.509	3.507	2.640	2.877
SWE13	3.274	2.317	2.728	3.593	4.011
SWE14	1.901	2.739	2.659	2.254	2.762

SWE15	2.837	2.859	3.359	3.206	3.287
SWE16	2.860	3.200	2.559	3.354	2.903
SWE17	2.813	3.091	2.626	2.663	3.233
SWE18	3.433	3.414	2.695	2.574	3.301
SWE19	3.351	3.399	2.731	2.999	3.676

Covariances

	SWE12	SWE13	SWE14	SWE15	SWE16
SWE12	9.319				
SWE13	2.961	9.076			
SWE14	2.827	2.874	5.404		
SWE15	3.595	3.524	2.940	8.282	
SWE16	2.390	2.955	3.048	2.577	7.277
SWE17	2.751	3.113	2.845	3.131	3.751
SWE18	3.106	2.804	2.791	3.328	3.867
SWE19	3.153	3.473	3.149	3.680	3.598

Covariances

	SWE17	SWE18	SWE19
SWE17	6.740		
SWE18	3.436	7.023	
SWE19	3.266	4.189	8.849

Correlations

	SWE2	SWE3	SWE4	SWE5	SWE6

SWE2	1.000				
SWE3	0.346	1.000			
SWE4	0.287	0.291	1.000		
SWE5	0.363	0.361	0.334	1.000	
SWE6	0.330	0.442	0.377	0.431	1.000
SWE7	0.346	0.337	0.405	0.371	0.477
SWE8	0.285	0.443	0.296	0.305	0.420
SWE9	0.335	0.431	0.298	0.367	0.399
SWE10	0.234	0.383	0.342	0.405	0.371
SWE11	0.357	0.441	0.304	0.377	0.373
SWE12	0.482	0.292	0.297	0.302	0.352
SWE13	0.323	0.346	0.370	0.341	0.369
SWE14	0.306	0.418	0.265	0.352	0.383
SWE15	0.319	0.334	0.304	0.362	0.372
SWE16	0.312	0.500	0.221	0.282	0.436
SWE17	0.390	0.547	0.253	0.268	0.368
SWE18	0.404	0.385	0.274	0.302	0.523
SWE19	0.359	0.387	0.326	0.350	0.441

Correlations

	SWE7	SWE8	SWE9	SWE10	SWE11
SWE7	1.000				
SWE8	0.374	1.000			
SWE9	0.361	0.423	1.000		
SWE10	0.393	0.301	0.342	1.000	
SWE11	0.443	0.391	0.434	0.324	1.000

SWE12	0.314	0.316	0.452	0.268	0.324
SWE13	0.366	0.295	0.357	0.370	0.458
SWE14	0.276	0.453	0.450	0.301	0.409
SWE15	0.332	0.382	0.459	0.346	0.393
SWE16	0.357	0.456	0.373	0.386	0.370
SWE17	0.365	0.457	0.398	0.318	0.429
SWE18	0.437	0.495	0.400	0.301	0.429
SWE19	0.380	0.439	0.361	0.313	0.425

Correlations

	SWE12	SWE13	SWE14	SWE15	SWE16
SWE12	1.000				
SWE13	0.322	1.000			
SWE14	0.398	0.410	1.000		
SWE15	0.409	0.406	0.439	1.000	
SWE16	0.290	0.364	0.486	0.332	1.000
SWE17	0.347	0.398	0.471	0.419	0.536
SWE18	0.384	0.351	0.453	0.436	0.541
SWE19	0.347	0.388	0.455	0.430	0.448

Correlations

	SWE17	SWE18	SWE19
SWE17	1.000		
SWE18	0.499	1.000	
SWE19	0.423	0.531	1.000

Model 4: operation

Mplus VERSION 8.3

MUTHEN & MUTHEN

02/09/2020 12:06 PM

INPUT INSTRUCTIONS

Title: CFA mit Dimension Repräsentation zur Überprüfung eindimensional_Kompetenz

Data: File is Daten_aufbereitet_CFA.dat;

Variable: Names are SWE1-SWE20;

usevariable SWE2-SWE19;

MODEL:

erstellen by SWE11 SWE2 SWE3 SWE12 SWE13 SWE15 SWE16 SWE17 SWE18 SWE19;

ablesen by SWE5 SWE4 SWE6 SWE7 SWE8 SWE9 SWE10 SWE14;

Analysis: type=general;

Estimator is MLR;

Output: sampstat standardized STDYX; modindices;

INPUT READING TERMINATED NORMALLY

SUMMARY OF ANALYSIS

Number of groups	1
Number of observations	376
Number of dependent variables	18
Number of independent variables	0
Number of continuous latent variables	2

Observed dependent variables

Continuous

SWE2 SWE3 SWE4 SWE5 SWE6 SWE7

SWE8 SWE9 SWE10 SWE11 SWE12 SWE13

SWE14 SWE15 SWE16 SWE17 SWE18 SWE19

Continuous latent variables

ERSTELLE ABLESEN

Estimator MLR

Information matrix OBSERVED

Maximum number of iterations 1000

Convergence criterion 0.500D-04

Maximum number of steepest descent iterations 20

Input data file(s)

Daten_aufbereitet_CFA.dat

Input data format FREE

SAMPLE STATISTICS

SAMPLE STATISTICS

Means

SWE2	SWE3	SWE4	SWE5	SWE6
7.519	8.231	5.689	6.311	6.255

Means

SWE7	SWE8	SWE9	SWE10	SWE11
5.218	7.702	8.106	6.388	7.697

Means

SWE12	SWE13	SWE14	SWE15	SWE16
7.207	6.404	8.258	7.428	7.144

Means

SWE17	SWE18	SWE19
7.662	7.221	6.133

Covariances

	SWE2	SWE3	SWE4	SWE5	SWE6
SWE2	7.686				
SWE3	2.359	6.040			
SWE4	2.496	2.245	9.821		
SWE5	3.264	2.877	3.397	10.523	
SWE6	2.578	3.066	3.335	3.944	7.956
SWE7	2.844	2.460	3.767	3.570	3.990
SWE8	2.059	2.838	2.418	2.579	3.081
SWE9	2.360	2.688	2.374	3.025	2.861
SWE10	2.091	3.038	3.453	4.233	3.369
SWE11	2.875	3.150	2.770	3.557	3.056
SWE12	4.081	2.194	2.841	2.986	3.032
SWE13	2.695	2.561	3.493	3.332	3.136
SWE14	1.970	2.387	1.929	2.656	2.511
SWE15	2.544	2.361	2.742	3.383	3.016
SWE16	2.335	3.315	1.872	2.466	3.314

SWE17	2.808	3.488	2.060	2.254	2.698
SWE18	2.965	2.510	2.279	2.594	3.906
SWE19	2.963	2.828	3.036	3.381	3.700

Covariances

	SWE7	SWE8	SWE9	SWE10	SWE11
SWE7	8.798				
SWE8	2.887	6.778			
SWE9	2.721	2.800	6.451		
SWE10	3.753	2.525	2.799	10.392	
SWE11	3.821	2.958	3.205	3.035	8.440
SWE12	2.846	2.509	3.507	2.640	2.877
SWE13	3.274	2.317	2.728	3.593	4.011
SWE14	1.901	2.739	2.659	2.254	2.762
SWE15	2.837	2.859	3.359	3.206	3.287
SWE16	2.860	3.200	2.559	3.354	2.903
SWE17	2.813	3.091	2.626	2.663	3.233
SWE18	3.433	3.414	2.695	2.574	3.301
SWE19	3.351	3.399	2.731	2.999	3.676

Covariances

	SWE12	SWE13	SWE14	SWE15	SWE16
SWE12	9.319				
SWE13	2.961	9.076			
SWE14	2.827	2.874	5.404		

SWE15	3.595	3.524	2.940	8.282	
SWE16	2.390	2.955	3.048	2.577	7.277
SWE17	2.751	3.113	2.845	3.131	3.751
SWE18	3.106	2.804	2.791	3.328	3.867
SWE19	3.153	3.473	3.149	3.680	3.598

Covariances

	SWE17	SWE18	SWE19
SWE17	6.740		
SWE18	3.436	7.023	
SWE19	3.266	4.189	8.849

Correlations

	SWE2	SWE3	SWE4	SWE5	SWE6
SWE2	1.000				
SWE3	0.346	1.000			
SWE4	0.287	0.291	1.000		
SWE5	0.363	0.361	0.334	1.000	
SWE6	0.330	0.442	0.377	0.431	1.000
SWE7	0.346	0.337	0.405	0.371	0.477
SWE8	0.285	0.443	0.296	0.305	0.420
SWE9	0.335	0.431	0.298	0.367	0.399
SWE10	0.234	0.383	0.342	0.405	0.371
SWE11	0.357	0.441	0.304	0.377	0.373
SWE12	0.482	0.292	0.297	0.302	0.352
SWE13	0.323	0.346	0.370	0.341	0.369

SWE14	0.306	0.418	0.265	0.352	0.383
SWE15	0.319	0.334	0.304	0.362	0.372
SWE16	0.312	0.500	0.221	0.282	0.436
SWE17	0.390	0.547	0.253	0.268	0.368
SWE18	0.404	0.385	0.274	0.302	0.523
SWE19	0.359	0.387	0.326	0.350	0.441

Correlations

	SWE7	SWE8	SWE9	SWE10	SWE11
SWE7	1.000				
SWE8	0.374	1.000			
SWE9	0.361	0.423	1.000		
SWE10	0.393	0.301	0.342	1.000	
SWE11	0.443	0.391	0.434	0.324	1.000
SWE12	0.314	0.316	0.452	0.268	0.324
SWE13	0.366	0.295	0.357	0.370	0.458
SWE14	0.276	0.453	0.450	0.301	0.409
SWE15	0.332	0.382	0.459	0.346	0.393
SWE16	0.357	0.456	0.373	0.386	0.370
SWE17	0.365	0.457	0.398	0.318	0.429
SWE18	0.437	0.495	0.400	0.301	0.429
SWE19	0.380	0.439	0.361	0.313	0.425

Correlations

	SWE12	SWE13	SWE14	SWE15	SWE16

SWE12	1.000				
SWE13	0.322	1.000			
SWE14	0.398	0.410	1.000		
SWE15	0.409	0.406	0.439	1.000	
SWE16	0.290	0.364	0.486	0.332	1.000
SWE17	0.347	0.398	0.471	0.419	0.536
SWE18	0.384	0.351	0.453	0.436	0.541
SWE19	0.347	0.388	0.455	0.430	0.448

Correlations

	SWE17	SWE18	SWE19
SWE17	1.000		
SWE18	0.499	1.000	
SWE19	0.423	0.531	1.000

Model 5: representation and context

Mplus VERSION 8.3

MUTHEN & MUTHEN

02/09/2020 12:09 PM

INPUT INSTRUCTIONS

Title: CFA mit Dimension Repräsentation zur Überprüfung zweidimensional_Gen_Repr_robust

Data: File is Daten_aufbereitet_CFA.dat;

Variable: Names are SWE1-SWE20;

MODEL:

Graph by SWE2 SWE5 SWE6 SWE8 SWE9 SWE12 SWE14 SWE15 SWE18 SWE19;

```
    Tabe by SWE3 SWE1 SWE4 SWE7 SWE10 SWE11 SWE13 SWE16 SWE17 SWE20;
    innermath by SWE4 SWE2 SWE5 SWE7 SWE9 SWE10 SWE11 SWE12 SWE13 SWE15;
    ausermath by SWE6 SWE1 SWE3 SWE8 SWE14 SWE16 SWE17 SWE18 SWE19 SWE20;
    Graph with Tabe@0;
    Graph with innermath@0;
    Graph with ausermath@0;
  Analysis: type=general;
        Estimator is MLR;
  Output: sampstat standardized STDYX; modindices;
INPUT READING TERMINATED NORMALLY
SUMMARY OF ANALYSIS
Number of groups                                    1
Number of observations                            376
Number of dependent variables                      20
Number of independent variables                     0
Number of continuous latent variables               4
Observed dependent variables
  Continuous
   SWE1       SWE2       SWE3       SWE4       SWE5       SWE6
   SWE7       SWE8       SWE9       SWE10      SWE11      SWE12
   SWE13      SWE14      SWE15      SWE16      SWE17      SWE18
   SWE19      SWE20
Continuous latent variables
   GRAPH      TABE       INNERMAT   AUSERMAT
Estimator                                         MLR
```

Information matrix OBSERVED

Maximum number of iterations 1000

Convergence criterion 0.500D-04

Maximum number of steepest descent iterations 20

Input data file(s)

Daten_aufbereitet_CFA.dat

Input data format FREE

SAMPLE STATISTICS

SAMPLE STATISTICS

Means

SWE1	SWE2	SWE3	SWE4	SWE5
7.207	7.519	8.231	5.689	6.311

Means

SWE6	SWE7	SWE8	SWE9	SWE10
6.255	5.218	7.702	8.106	6.388

Means

SWE11	SWE12	SWE13	SWE14	SWE15
7.697	7.207	6.404	8.258	7.428

Means

SWE16	SWE17	SWE18	SWE19	SWE20
7.144	7.662	7.221	6.133	8.899

Covariances

	SWE1	SWE2	SWE3	SWE4	SWE5
SWE1	7.824				
SWE2	0.834	7.686			
SWE3	2.973	2.359	6.040		
SWE4	2.192	2.496	2.245	9.821	
SWE5	2.345	3.264	2.877	3.397	10.523
SWE6	2.734	2.578	3.066	3.335	3.944
SWE7	2.388	2.844	2.460	3.767	3.570
SWE8	2.937	2.059	2.838	2.418	2.579
SWE9	2.028	2.360	2.688	2.374	3.025
SWE10	2.834	2.091	3.038	3.453	4.233
SWE11	2.839	2.875	3.150	2.770	3.557
SWE12	1.561	4.081	2.194	2.841	2.986
SWE13	1.919	2.695	2.561	3.493	3.332
SWE14	2.058	1.970	2.387	1.929	2.656
SWE15	2.153	2.544	2.361	2.742	3.383
SWE16	2.781	2.335	3.315	1.872	2.466
SWE17	2.400	2.808	3.488	2.060	2.254
SWE18	2.191	2.965	2.510	2.279	2.594
SWE19	2.143	2.963	2.828	3.036	3.381
SWE20	1.449	1.611	2.295	1.312	1.273

Covariances

	SWE6	SWE7	SWE8	SWE9	SWE10

SWE6	7.956				
SWE7	3.990	8.798			
SWE8	3.081	2.887	6.778		
SWE9	2.861	2.721	2.800	6.451	
SWE10	3.369	3.753	2.525	2.799	10.392
SWE11	3.056	3.821	2.958	3.205	3.035
SWE12	3.032	2.846	2.509	3.507	2.640
SWE13	3.136	3.274	2.317	2.728	3.593
SWE14	2.511	1.901	2.739	2.659	2.254
SWE15	3.016	2.837	2.859	3.359	3.206
SWE16	3.314	2.860	3.200	2.559	3.354
SWE17	2.698	2.813	3.091	2.626	2.663
SWE18	3.906	3.433	3.414	2.695	2.574
SWE19	3.700	3.351	3.399	2.731	2.999
SWE20	1.169	1.339	1.659	1.960	1.217

Covariances

	SWE11	SWE12	SWE13	SWE14	SWE15
SWE11	8.440				
SWE12	2.877	9.319			
SWE13	4.011	2.961	9.076		
SWE14	2.762	2.827	2.874	5.404	
SWE15	3.287	3.595	3.524	2.940	8.282
SWE16	2.903	2.390	2.955	3.048	2.577
SWE17	3.233	2.751	3.113	2.845	3.131

SWE18	3.301	3.106	2.804	2.791	3.328
SWE19	3.676	3.153	3.473	3.149	3.680
SWE20	1.895	1.792	1.365	1.696	1.948

Covariances

	SWE16	SWE17	SWE18	SWE19	SWE20
SWE16	7.277				
SWE17	3.751	6.740			
SWE18	3.867	3.436	7.023		
SWE19	3.598	3.266	4.189	8.849	
SWE20	2.065	2.280	1.714	1.359	4.958

Correlations

	SWE1	SWE2	SWE3	SWE4	SWE5
SWE1	1.000				
SWE2	0.108	1.000			
SWE3	0.433	0.346	1.000		
SWE4	0.250	0.287	0.291	1.000	
SWE5	0.258	0.363	0.361	0.334	1.000
SWE6	0.347	0.330	0.442	0.377	0.431
SWE7	0.288	0.346	0.337	0.405	0.371
SWE8	0.403	0.285	0.443	0.296	0.305
SWE9	0.286	0.335	0.431	0.298	0.367

SWE10	0.314	0.234	0.383	0.342	0.405
SWE11	0.349	0.357	0.441	0.304	0.377
SWE12	0.183	0.482	0.292	0.297	0.302
SWE13	0.228	0.323	0.346	0.370	0.341
SWE14	0.317	0.306	0.418	0.265	0.352
SWE15	0.267	0.319	0.334	0.304	0.362
SWE16	0.369	0.312	0.500	0.221	0.282
SWE17	0.330	0.390	0.547	0.253	0.268
SWE18	0.296	0.404	0.385	0.274	0.302
SWE19	0.257	0.359	0.387	0.326	0.350
SWE20	0.233	0.261	0.419	0.188	0.176

Correlations

	SWE6	SWE7	SWE8	SWE9	SWE10
SWE6	1.000				
SWE7	0.477	1.000			
SWE8	0.420	0.374	1.000		
SWE9	0.399	0.361	0.423	1.000	
SWE10	0.371	0.393	0.301	0.342	1.000
SWE11	0.373	0.443	0.391	0.434	0.324
SWE12	0.352	0.314	0.316	0.452	0.268
SWE13	0.369	0.366	0.295	0.357	0.370
SWE14	0.383	0.276	0.453	0.450	0.301
SWE15	0.372	0.332	0.382	0.459	0.346
SWE16	0.436	0.357	0.456	0.373	0.386
SWE17	0.368	0.365	0.457	0.398	0.318

SWE18	0.523	0.437	0.495	0.400	0.301
SWE19	0.441	0.380	0.439	0.361	0.313
SWE20	0.186	0.203	0.286	0.347	0.170

Correlations

	SWE11	SWE12	SWE13	SWE14	SWE15
SWE11	1.000				
SWE12	0.324	1.000			
SWE13	0.458	0.322	1.000		
SWE14	0.409	0.398	0.410	1.000	
SWE15	0.393	0.409	0.406	0.439	1.000
SWE16	0.370	0.290	0.364	0.486	0.332
SWE17	0.429	0.347	0.398	0.471	0.419
SWE18	0.429	0.384	0.351	0.453	0.436
SWE19	0.425	0.347	0.388	0.455	0.430
SWE20	0.293	0.264	0.204	0.328	0.304

Correlations

	SWE16	SWE17	SWE18	SWE19	SWE20
SWE16	1.000				
SWE17	0.536	1.000			
SWE18	0.541	0.499	1.000		
SWE19	0.448	0.423	0.531	1.000	
SWE20	0.344	0.394	0.290	0.205	1.0
